# Diversity and correlation analysis of different root exudates on the regulation of microbial structure and function in soil planted with *Panax notoginseng*

**DOI:** 10.3389/fmicb.2023.1282689

**Published:** 2023-12-06

**Authors:** Huineng Shi, Jianli Yang, Qi Li, Cier PinChu, Zhanhua Song, Honglei Yang, Yu Luo, Chunlan Liu, Wei Fan

**Affiliations:** ^1^State Key Laboratory of Conservation and Utilization of Bio-Resources in Yunnan, The Key Laboratory of Medicinal Plant Biology of Yunnan Province, National and Local Joint Engineering Research Center on Germplasm Innovation and Utilization of Chinese Medicinal Materials in Southwest China, Yunnan Agricultural University, Kunming, China; ^2^College of Resources and Environment, Yunnan Agricultural University, Kunming, China; ^3^College of Landscape and Horticulture, Yunnan Agricultural University, Kunming, China; ^4^College of Food Science and Technology, Yunnan Agricultural University, Kunming, China

**Keywords:** root exudates, macrogenome, *Panax notoginseng* planting soil, microbial community structure, functional genes

## Abstract

**Introduction:**

Specific interactions between root exudates and soil microorganisms has been proposed as one of the reasons accounting for the continuous cropping obstacle (CCO) of *Panax notoginseng*. However, rotation of other crops on soils planted with *P. notoginseng* (SPP) did not show CCO, suggesting that root exudates of different crops differentially regulate soil microorganisms in SPP.

**Methods:**

Here, we investigated the microbial community structure and specific interaction mechanisms of the root exudates of the four plant species, *P. notoginseng* (Pn), *Zea mays* (Zm), *Nicotiana tabacum* (Nt) and *Perilla frutescens* (Pf), in SPP by static soil culture experiment.

**Results:**

The results showed that the chemical diversity of root exudates varied significantly among the four plant species. Pn had the highest number of unique root exudates, followed by Nt, Zm and Pf. Terpenoids, flavonoids, alkaloids and phenolic acids were the most abundant differentially accumulated metabolites (DAMs) in Pn, Nt, Zm and Pf, respectively. However, lipids were the most abundant common DAMs among Zm Nt and Pf. Pn root exudates decreased the relative abundance of bacteria, but increased that of fungi. While specific DAMs in Pn enriched *Phenylobacterium_zucineum*, *Sphingobium_yanoikuyae*, *Ophiostoma_ulmi* and functional pathways of Nucleotide excision repair, Streptomycin biosynthesis, Cell cycle-Caulobacter and Glycolysis/Gluconeogenesis, it inhibited *Paraburkholderia _caledonica* and *Ralstonia_pickettii*. However, common DAMs in Zm, Nt and Pf had opposite effects. Moreover, common DAMs in Zm, Nt and Pf enriched *Ralstonia_pseudosolanacearum* and functional pathway of Xylene degradation; unique DAMs in Zm enriched *Talaromyces_purcureogeneus*, while inhibiting *Fusarium_tricinctum* and functional pathways of Nucleotide excision repair and Alanine, aspartate and glutamate metabolism; unique DAMs in Pf enriched *Synchytrium_taraxaci*.

**Discussion:**

The core strains identified that interact with different root exudates will provide key clues for regulation of soil microorganisms in *P. notoginseng* cultivation to alleviate CCO.

## 1 Introduction

Continuous cropping obstacle (CCO) is common in agricultural production, and about 70% of cultivated tuberous medicinal plants have some degree of CCO (Wu and Lin, 2020). *Panax notoginseng*, as one of the most famous Chinese traditional medicinal plants belonging to the Panax genus in the Araliaceae family, shows particularly serious CCO during its planting process, as manifested by the fact that the fallow and rotation period for replanting *P. notoginseng* is more than 15 years (Yang et al., 2018). Previous studies have attributed the CCO of *P. notoginseng* to the imbalance of soil nutrients, deterioration of soil physical, allelopathic autotoxicity, and microbial community imbalance (Zhang et al., 2019; Ye et al., 2021; Zhang et al., 2021). Especially, the soil microbial structure continuously shifts from “bacterial type” to “fungal type” during the 3-years planting cycle of *P. notoginseng*, thus exacerbating negative plant–soil feedback (Dong et al., 2016; Wei et al., 2018; Luo et al., 2019). However, the mechanism underlying the change of soil microbial structure in *P. notoginseng* remains unclear.

Root exudates are intermediator between plants and microorganisms, and play a crucial role in responding to environmental changes in ecosystems (Bais et al., 2006; Williams and Vries, 2019). Root exudates mainly mediate plant–soil-microorganism interactions to build the soil microbiome and function (Dennis et al., 2010; Chaparro et al., 2013; Carvalhais et al., 2015). However, the interactions have both beneficial and harmful effects. According to the previous reports, flavones secreted by *Zea mays* promote the enrichment of Oxalobacteraceae in the rhizosphere, thereby improving plant growth and nitrogen acquisition (Yu et al., 2021). Glutamic acid in root exudates of *Fragaria ananassa* controls fusarium wilt on the root by recruiting *Streptomyces* (Kim et al., 2021). In contrast, cinnamic, myristic and fumaric acid in root exudates of *Nicotiana tabacum* can be used as chemoattractants to induce the infection and colonization of plants by *Ralstonia solanacearum* (Li et al., 2016). Meanwhile, it has been reported that cinnamic also induces oxidative stress in *Cucumis sativus* roots, thus promoting the incidence rate of fusarium wilt (Ye et al., 2004). Therefore, root exudates play a dual roles in soil, either recruiting beneficial bacteria to promote plant growth or inducing pathogenesis to cause plant disease and death.

The occurrence of root rot is a major manifestation of CCO in *P. notoginseng*. Previous studies have found that ginsenosides and phenols secreted by *P. notoginseng* roots into the growth medium can interact with rhizosphere microbiota (Luo et al., 2020, 2021; Bao et al., 2022). On the one hand, root rot-infected *P. notoginseng* rhizosphere harbors dynamically pathogenic microbiota driven by the shift of phenolic acids (Wang et al., 2021). In addition, reducing phenols can decrease the occurrence of root rot by reducing the stimulation of pathogenic fungi and the pressure to inhibit beneficial bacteria. On the other hand, high concentration of autotoxic ginsenoside Rg1 and its cell wall degradation products in *P. notoginseng* can alter the structure and function of rhizosphere microbiotas, thereby promoting the growth of *Fusarium oxysporum* and exacerbating root rot (Luo et al., 2020; Xu et al., 2021). In contrast, appropriate concentration of ginsenoside Rb1, Rg1 and Rd. can alleviate negative plant–soil feedback by enriching Burkholderia to degrade autotoxic ginsenoside and antagonize soil-borne pathogens (Luo et al., 2021). These studies have revealed that the root exudates of *P. notoginseng* can modulate the soil microbial community, partially explaining the cause of CCO. However, complete degradation of root exudates of *P. notoginseng* in soil does not take a long time, and replanting *P. notoginseng* after many years can still produce CCO. Does this imply that there is a specific regulatory relationship between root exudates and pathogenic microbes in soil planted with *P. notoginseng* (SPP)?

In agricultural production, rotation with *N. tabacum* and *Z. mays* in SPP does not exhibit CCO. Similarly, rotation of *Perilla frutescens* after planting *Pseudostellaria heterophylla* and *Panax quinquefolius*, two *Panax* species, displayed alleviation of CCO (Zhao et al., 2005; Lin et al., 2018). Therefore, comparing the differences in root exudates and regulation microbial communities between *P. notoginseng* and other plants in SPP that has been lie fallow for a period of time when ginsenosides have been completely degraded will help to further unravel the regulatory mechanisms by which root exudates of *P. notoginseng* specifically drives the changes in soil microbial communities.

In this study, we modeled plant–soil microbial interactions in a static soil culture experiment based on SPP with exogenous addition of root exudates of *P. notoginseng*, *Z. mays*, *N. tabacum* and *P. frutescens*. We hypothesized that (a) differences in root exudates between *P. notoginseng* and other plants are a prerequisite for CCO; (b) The unique root exudates of *P. notoginseng* can induce microbial community imbalance by stimulating the accumulation of pathogenic bacteria and inhibiting the growth of beneficial bacteria in the SPP, which can be ameliorated by the unique and common root exudates secreted by *Z. mays*, *N. tabacum* and *P. frutescens*; (c) The changes in soil microbial structure and diversity induced by differental root exudates reflect differences in soil microbial function.

## 2 Materials and methods

### 2.1 Soil sample collection

The SPP was collected from Shilin County, Yunnan Province (latitude 24°48′16″N, longitude 103°26′12″W, altitude 1875 m), which had been left fallow for 2 years after planting *P. notoginseng* for 3 years. Tillage soil (0 to 20 cm) was collected, sieved (2 mm mesh) and then stored at 4°C until use. Soil physicochemical properties: pH 5.46; organic matter 16.21 g/kg; total nitrogen 0.39 g/kg; total phosphorus 1.78 g/kg; total potassium 14.53 g/kg; alkaline hydrolyzed nitrogen 58.63 mg/kg; available phosphorus 1.33 mg/kg; available potassium 84.59 mg/kg. PPS had CCO effect for replanting *P. notoginseng*, but no triterpenoid saponins were detected, indicating that remaining traces of root exudates from the previous *P. notoginseng* had disappeared (Supplementary Figure S1), which is suitable for carrying out research on the effects of different root exudates on the original microbial communities in SPP.

### 2.2 Plant materials and cultivation

All plant materials were grown in an artificial climate incubator. *Z. mays* was cultured in Hoagland nutrient solution (pH5.8), whereas *P. notoginseng*, *N. tabacum* and *P. frutescens* were cultured in 1/5 Hoagland nutrient solution (pH5.8 for *P. notoginseng*, pH5.8 for *N. tabacum* and *P. frutescens*). Firstly, *Z. mays* seeds were sowed in a seedling trays containing sterilized vermiculite for germination, while *N. tabacum* and *P. frutescens* seeds were placed in plastic trays lined with gauze for germination. The seedling trays and gauze were kept moist throughout the germination process. One week after germination, seedlings of *Z. mays*, *N. tabacum* and *P. frutescens* were transplanted into 8 L-plastic pots. The cultivation conditions: 16 h/28°C day (light intensity of 18,000 Lx), 8 h/21°C night, and a humidity of 60%. 2-years old *P. notoginseng* plants were collected from Shilin County, Yunnan Province (latitude 24°41′32″N, longitude 103°39′3″W, altitude 1940 m). The soil was washed from the roots with deionized water and then transplanted into 8 L-plastic pots. The cultivation conditions:16 h/23°C day (light intensity of 3,000 Lx), 8 h/18°C night, and a humidity of 70%. The solution was renewed every 4 days.

### 2.3 Collection of root exudates and widely targeted metabolome detection

For collection of root exudates, after cultured for 30, 60, and 90 days, the plant roots of *Z. mays*, *N. tabacum* and *P. frutescens* were rinsed 3–4 times with sterile water and collected in sealed opaque plastic bags containing 500 mL of sterile water for 24 h. However, for *P. notoginseng*, after cultured for 14 days, the roots were collected as the same way. The collected root exudates were filtered through a 0.22 μm filter membrane and dried using a vacuum freeze dryer. The freeze-dried root exudates were stored at −80°C. Dissolved organic carbon (DOC) was utilized for quantitative analysis of different plant root exudates (Liao et al., 2021).

Root exudates were dissolved in 1.2 mL of 70% methanol extract and vortexed for 30 s every 30 min for six times. The samples w were placed in a refrigerator at 4°C overnight. The next day, the extract was centrifuged at 12000 rpm for 10 min, and the supernatant was filtered using a 0.22 μm filter membrane. The metabolites in the extracts of each sample were analyzed by ultra performance liquid chromatography–tandem mass spectrometry (UPLC-MS/MS) (Li M. et al., 2022).

### 2.4 Static cultivation of soil with the addition of root exudates

Prior to the addition of root exudates, sieved (2 mm) SPP was pre-incubated in a dark artificial climate chamber at 20°C for 2 weeks at a constant (35%) moisture content to restore soil microbial activity and stabilize the soil microbial community. After pre-cultivation, 200 g of SPP (dry weight equivalent) was weighed into sterile plastic culture bottles, respectively. The experiment was set up with five treatments of CK (Sterile water), Pn (*P. notoginseng* root exudates) Zm (*Z. mays* root exudates), Nt (*N. tabacum* root exudates) and Pf (*P. frutescens* root exudates), and each treatment consisted of five biological replicates. The total concentration of 4 monomeric saponins, i.e., R1, Rg1, Re and Rd., in Pn root exudates was found to be 20 μg mL^−1^. Based on this, root exudates of Pn were added into soil every 5 days for a total of 5 times, and the final total concentration of 4 monomeric saponins (R1, Rg1, Re and Rd) was less than 1 μg g^−1^ dry soil. This concentration is less than the saponin autotoxic concentration for the normal growth of *P. notoginseng* (Yang et al., 2015). In addition, the amount of root exudates added by Pn is used to calculate the added amount of Zm, Nt, and Pf root exudates. All soil samples were incubated at a constant (35%) moisture content in the dark at 20°C throughout the incubation period. After 30 days of treatment, soil samples were collected for determination of microbial activity, abundance and community composition.

### 2.5 Metagenomic sequencing and analysis

A DNeasy PowerSoil Pro Kit (Qiagen©, USA) was used to extract genomic DNA from soil samples following the manufacturer’s instructions. NanoDrop 2000 spectrophotometer (Thermo Scientific, Waltham, MA, United States) was used to estimate the concentration and purification of soil DNA, and DNA quality was checked by 1% agarose gel electrophoresis. For the library preparation, a total of 1 μg DNA from each sample was used. Sequencing libraries were constructed using NEBNext UltraDNA Library Prep Kit for Illumina (NEB, Ipswich, MA, USA) following the manufacturer’s instructions. Briefly, the DNA samples were fragmented to a size of about 350 bp by sonication, then DNA fragments underwent end-repair, A-tailing, and adapter ligation, purification and PCR amplification for Illumina sequencing. Finally, PCR products were purified (AMPure XP system, Beckman Coulter, Brea, CA, USA) and libraries were analyzed for size distribution by Agilent 2,100 Bioanalyzer (Agilent Technologies, Palo Alto, CA, USA) and quantified using real-time PCR. The clustering of the index-coded samples was performed on a cBot Cluster Generation System (Illumina, San Diego, CA, USA) according to the manufacturer’s instructions. After cluster generation, the library preparations were sequenced on an Illumina PE150 platform and 150 bp paired-end reads were generated.

After sequencing, the original sequence data was controlled for quality. Low-quality sequences (length below 150 bp, average Phred scores <20, mononucleotide repeats over 8 bp, and with ambiguous bases), and n-containing and contaminated reads were removed from the data, resulting in better quality assembled sequences. Open reading frame (ORF) prediction of contigs in the assembly results were obtained using MetaGene. Genes with nucleic acid length greater than or equal to 100 bp were selected and translated into amino acid sequences. Nonredundant gene sets were constructed by clustering using CD-HIT 4.5.6 software, and SOAPaligner 2.21 software, was used to compare the high-quality reads of each sample with the non-redundant gene set to calculate the abundance of the gene in the corresponding sample. Subsequently, BLASTP 2.6.0 alignment of non-redundant gene sets with Non-Redundancy (NR), Evolutionary genealogy of genes: Nonsupervised Orthologous Groups (EggNOG), and Kyoto Encyclopedia of Genes and Genomes (KEGG) databases was performed using DIAMOND v 0.8.24.86 software. Species annotations from the taxonomic information database corresponding to the NR database were obtained, and statistical species at each taxonomic level, such as phylum, genus, and species were constructed. Quantitative macrogenomics analysis was performed by Micromax Technology Group Limited (Shenzhen, China), and metagenomic data were integrated to construct a network diagram of key microbial and root exudates compounds using the KEGG database and species annotation abundance.

### 2.6 Determination of soil enzymatic activities

Soil urease activity was determined by colorimetric analysis of sodium phenol-sodium hypochlorite (Kandeler and Gerber, 1988; Van Wyk et al., 2017). Soil sucrase, cellulase, acid phosphatase and polyphenol oxidase activities were determined by colorimetric method (Guan, 1986). Soil catalase activity was determined by potassium permanganate titration (Sinha, 1972).

### 2.7 Statistic analysis

SPSS version 20.0 statistical software was used to statistically analyze the data. Mean separation between treatments was analyzed by one-way analysis of variance (ANOVA) and Duncan multiple range test (*p* < 0.05). For UPLC-MS/MS data of root exudates, principal coordinate analysis (PcoA) was performed based on Bray Curtis (Huerta-Cepas et al., 2015; Kanehisa et al., 2017). Upset plots of metabolite intersections for differences in relative abundance of root exudates of four plant species were plotted by using the R software (version 3.6.3) UpSetR package. Random forest analysis of the effects of chemodiversity of root exudates and soil enzyme activities on microbial diversity was carried out by using the “randomForesi” program package in R. Beta diversity based on Bray-Curtis metrics was used to compare the differences between different treatment groups of bacteria and fungi byPCoA (Liu et al., 2020). Common or unique taxa at each phylogenetic level and functional pathways were visualized between groups using the R package VennDiagram. Linear discriminant analysis (LDA) effect sizes (LEfSe) were further applied to identify bacterial and fungal taxa that were significantly differentially enriched between treatment groups as well as statistically differentiated functional pathways (Segata et al., 2011). In order to find out the relationship of differential root exudates with soil bacteria and fungi as well as functional pathways, spearman correlation calculations were carried out using R version (3.6.3), and then network visualization analysis of root exudates with soil bacteria and fungi as well as functional pathways was constructed by igraph package (1.2.6).

## 3 Results

### 3.1 Changes in the metabolic profile of root exudates

**Figure 1:**
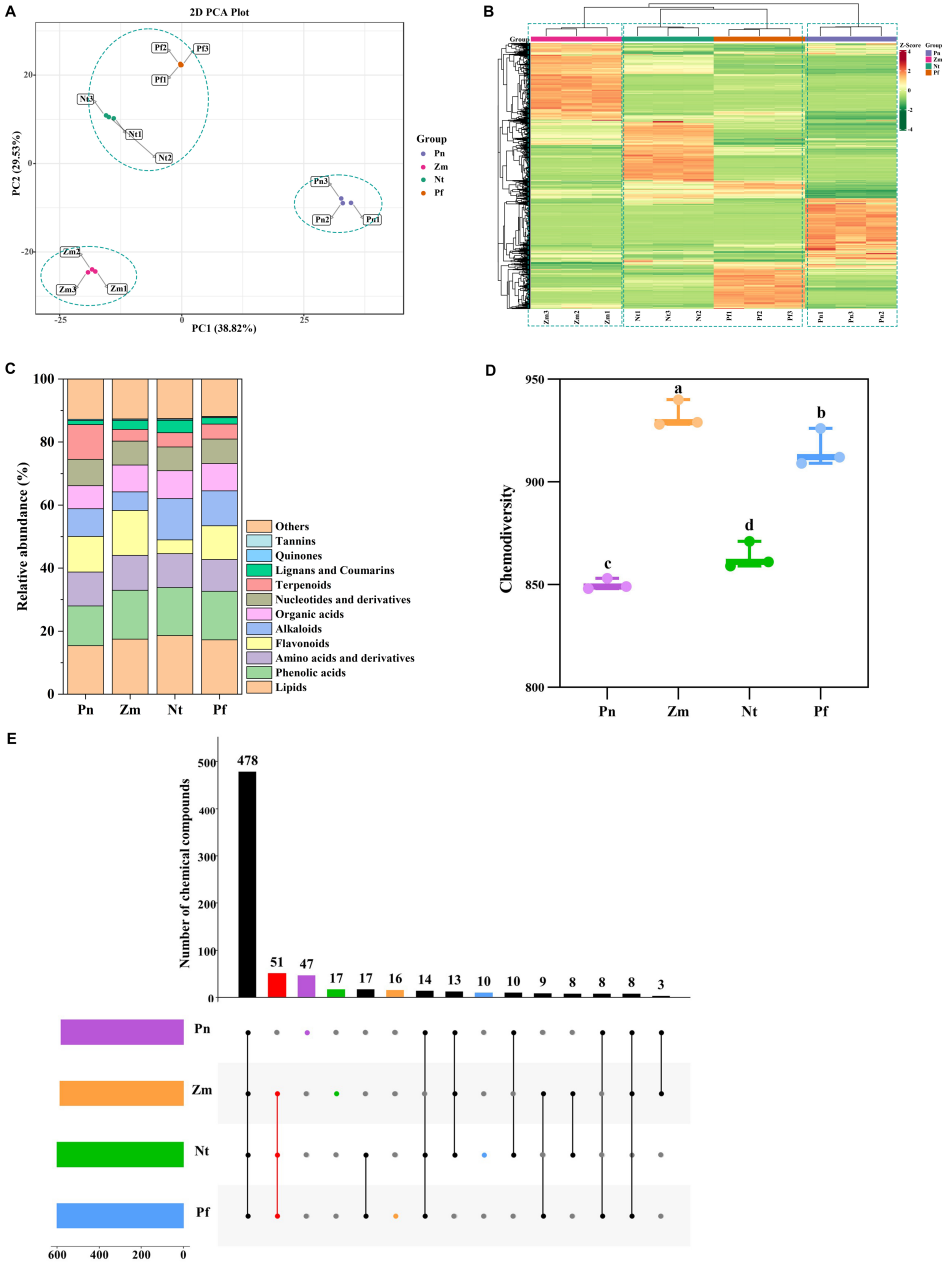
Multivariate statistical analysis of metabolomics data on root exudates of four plant species. **(A)** PCA score plot. **(B)** Cluster analysis. **(C)** Relative abundance of metabolite composition. **(D)** Chemodiversity of metabolites. **(E)** Numbers of differential accumulated metabolites (DAMs). Boxes with the same lowercase letter indicate no significant difference between treatments based on the LSD test (*p* < 0.05).

The compound composition of the root exudates of Pn, Zm, Nt, and Pf was investigated using UPLC-MS/MS by which a total of 1,179 metabolites were detected (Supplementary Table S1). Principal component analysis (PCA) revealed that three biological replicates of each group were closely gathered together, indicating that the metabolome data were reproducible and reliable (Figure 1A). Hierarchical clustering showed that these metabolites were classified into three groups, with Nt and Pf clustered into the first group, Zm the second, and Pn the third (Figure 1B). Metabolites detected included phenolic acids, amino acids and their derivatives, nucleotides and their derivatives, flavonoids, quinones, lignans and coumarins, others, alkaloids, terpenoids, organic acids, lipids, and tannins (Figure 1C). Among them, the amounts of each category in four plant metabolites varied little, showing the highest amount of lipids and the lowest amount of quinones and tannins (Supplementary Table S2). In addition, there were significant differences in the chemical diversity of the four plant metabolites (Figure 1D). The Venn diagram showed that Pn, Zm, Nt and Pf contained 47, 16, 17 and 10 unique differentially accumulated metabolites (DAMs), respectively, while Zm, Nt and Pf shared 51 common DAMs (Figure 1E).

We further counted unique and common DAMs in the root exudates of the four plant species. The results showed that unique DAMs in Pn were mainly dominated by Terpenoids (32), followed by Flavonoids (7) (Supplementary Figure S2A and Supplementary Table S3); unique DAMs in Zm were mainly dominated by Flavonoids (6), followed by Phenolic acids (3) (Supplementary Figure S2B and Supplementary Table S4); unique DAMs in Nt were mainly dominated by Alkaloids (7), followed by Lignans and Coumarins (4) (Supplementary Figure S2C and Supplementary Table S5); unique DAMs in Pf were mainly dominated by Phenolic acids (3) and Others (3), followed by Flavonoids (2) (Supplementary Figure S2D and Supplementary Table S6). In comparison, common DAMs in the root exudates of Zm, Nt and Pf included Lipids (17), Phenolic acids (11), Alkaloids (7), Organic acids (7), Others (4), Flavonoids (2), Lignans and Coumarins (1), Tannins (1), Terpenoids (1) (Supplementary Figure S3 and Supplementary Table S7). Taken together, the root exudates of the four plant species differed both in kinds and contents, with Pn having the highest number of unique DAMs, followed by Nt, Zm and Pf.

### 3.2 Illumina sequencing data analysis

As shown in Supplementary Table S8, Illumina sequencing data analysis revealed that a total of 568,793,566 raw reads were obtained from 25 libraries, in which 531,171,282 represent clean reads. The total number of bases in the sequencing raw data was 170.62 GB, and the percentage of the number of G/C bases to the total number of bases was 61.80%, and the number of sequences remaining after filtering to the average percentage of raw reads was 93.33%. The average proportion of raw and filtered bases with quality scores higher than 20 was 91.50 and 94.24%, respectively, and the average proportion of raw and filtered bases with quality scores higher than 30 was 96.61 and 98.36%, respectively. The amount of sequencing data of all samples was greater than 5 G, and some samples could be close to 10 G, which showed that the quality of raw data was high. In addition, the non-redundant gene catalogs of bacteria and fungi were constructed, and 56,980,744 and 891,472 cataloged genes were obtained, respectively.

### 3.3 Bacterial community composition of SPP with added root exudates

**Figure 2:**
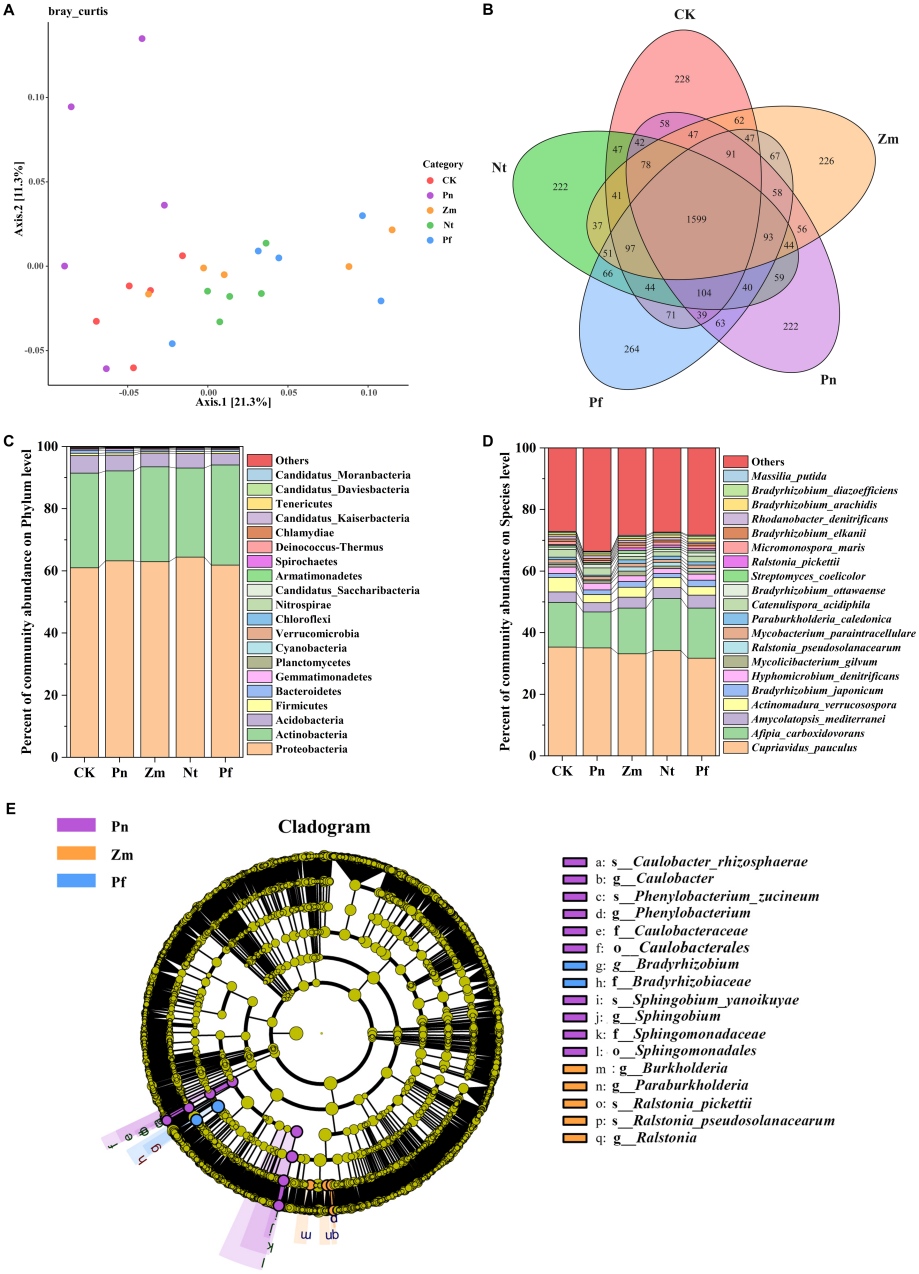
Effects of different root exudates on the composition of bacterial taxa in SPP. **(A)** Bray Curtis-PCoA plot. **(B)** Venn diagram. **(C)** Bacterial community structure at the gate level. **(D)** Bacterial community composition at the species level. **(E)** Evolutionary map of bacterial taxa (LDA ≥ 3.5) based on LEfSe. Phylum or species with mean RA < 1% were combined and labeled as “Others”.

The PcoA of soil bacterial community in SSP by adding different root exudates showed that two principal coordinates explained 32.60% of the microbial community variation in all the samples, with Axis.1 explaining 21.30% of the variation and Axis.2 explaining 11.30% of the variation (Figure 2A). Meanwhile, all the samples were divided into five groups, in which Pn was close to the principal coordinates, Nt, Pf and Zm had similar bacterial community structure and were far away from the principal coordinates, and CK was among Pn and Nt, Pf and Zm (Figure 2A). This indicated that the bacterial community structure of SSP was susceptible to the easily affected by root exudates, and there were significant differences in the bacterial community structure in SSP after treatment with Pn, Nt, Pf and Zm.

Macrogenomic sequencing detected a total of 4,263 species from the five soil samples, of which 288, 222, 226, 222 and 264 unique species as well as 1,599 common species were detected in CK, Pn, Zm, Nt and Pf, respectively (Figure 2B). At the phylum level, the dominant bacterial phyla in SPP were Proteobacteria, Actinobacteria and Acidobacteria (Relative Abundance (RA), RA > 1%), and the total number of these phyla accounted for 97.52% of the bacterial composition, with Proteobacteria (62.68%) and Acidobacteria (30.14%) accounted for the largest proportion (Figure 2C). Compared with CK, Pn, Zm, Nt and Pf increased RA of Proteobacteria but decreased RA of Acidobacteria, while Zm and Pf increased RA of Actinobacteria (Figure 2C). At the species level, we analyzed the top 20 bacteria in terms of RA. The results showed that *Afipia_carboxidovorans*, *Amycolatopsis_mediterranei*, *Mycolicibacterium_gilvum*, *Ralstonia_pseudosolanacearum*, *Paraburkholderia_caledonica*, *Streptomyces_coelicolor*, *Ralstonia_pickettii*, *Micromonospora_maris* and *Massilia_putida* had lower RA in Pn than Zm, Nt and Pf, while *Cupriavidus_pauculus* and *Hyphomicrobium_denitrificans* had higher re RA in Pn than Zm, Nt and Pf (Figure 2D).

LEfSe analysis was utilized in order to identify bacteria taxa with significant abundance differences (LDA ≥ 3.5) in SPP after each treatment. The results showed that there were 17 significant taxonomic bacterial groups in the five treatments, of which the taxa significantly enriched in Pn were *Caulobacterales, Caulobacteraceae, Caulobacter, Caulobacter_rhizosphaerae, Sphingomonadales, Sphingomonadaceae, Sphingobium, Sphingobium_yanoikuyae, Phenylobacterium, and Phenylobacterium_zucineum*; taxa significantly enriched in Zm were *Ralstonia, Ralstonia_pickettii, Ralstonia_pseudosolanacearum, Paraburkholderia and Burkholderia*; and taxa significantly enriched in Pf were *Bradyrhizobiaceae* and *Bradyrhizobium* (Figure 2E).

### 3.4 Fungal community composition of SPP with added root exudates

**Figure 3:**
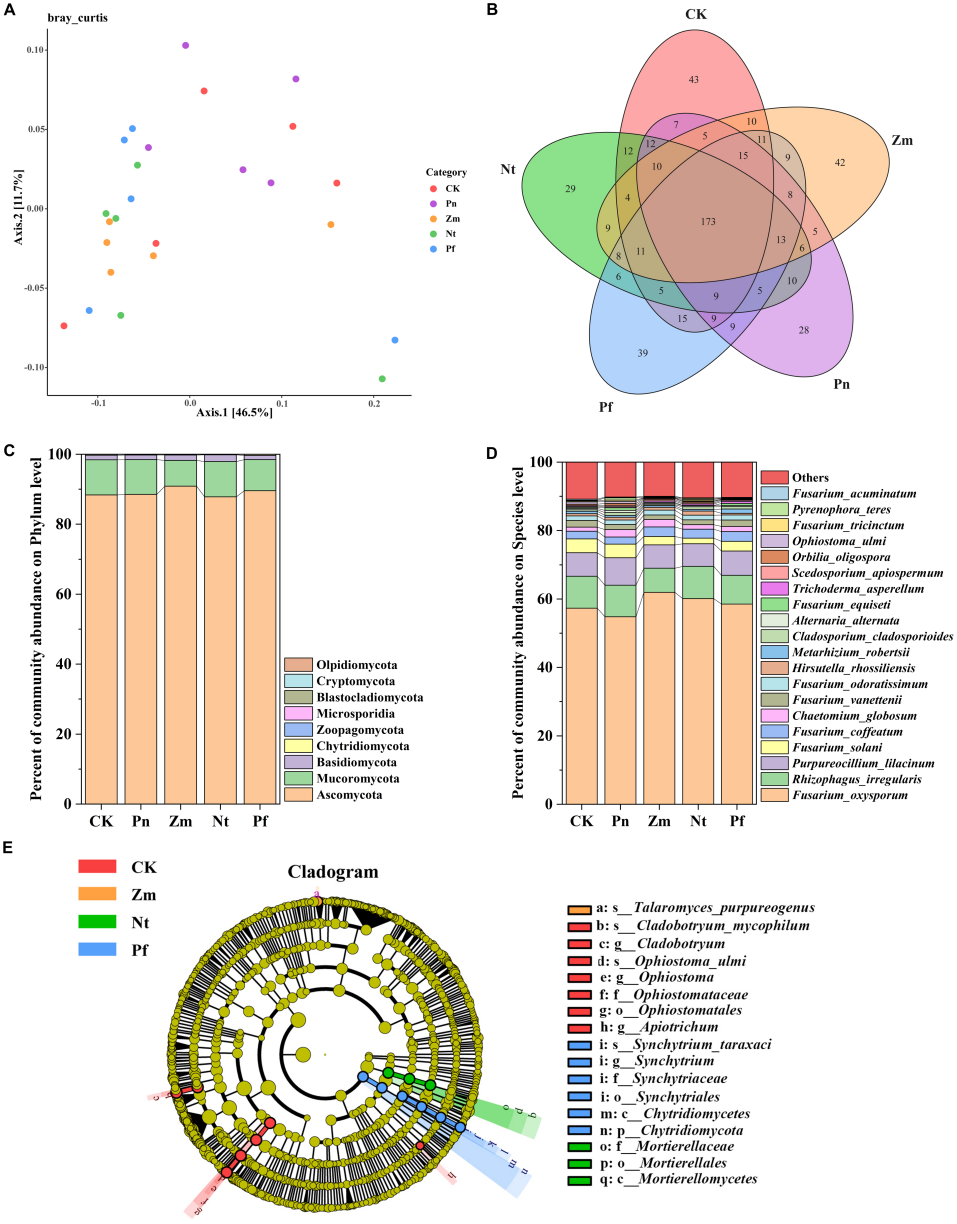
Effects of different root exudates on the composition of fungal taxa in SPP. **(A)** Bray Curtis-PCoA plot. **(B)** Venn diagram. **(C)** Fungal community structure at the gate level. **(D)** Fungal community composition at the species level. **(E)** Evolutionary map of fungal taxa (LDA ≥3) based on LEfSe. Species with mean RA <1% were combined and labeled as “Others”.

The PcoA of soil fungal community in SSP by adding different root exudates showed that two principal coordinates explained 58.20% of the variation in fungal community, with Axis.1 explaining 46.50% of the variation and Axis.2 explaining 11.70% of the variation (Figure 3A). Meanwhile, the fungal composition of Pn was different from that of Nt, Pf and Zm, where Nt and Pf had some similarities (Figure 3A).

Macrogenomic sequencing detected a total of 577 species from the five soil samples, of which 43, 28, 42, 29 and 39 unique species as well as 173 common species were detected in CK, Pn, Zm, Nt and Pf, respectively (Figure 3B). At the phylum level, the dominant bacterial phyla in SPP were Ascomycota, Mucoromycota and Basidiomycota(RA >1%), and the total number of these phyla accounted for 97.52% of the fungal composition, with Ascomycota (89.05%) accounting for the largest proportion, followed by Mucoromycota (9.26%) (Figure 3C). Compared with CK, Pn decreased RA of Ascomycota but increased RA of Mucoromycota, while the opposite was true for the Zm, Nt and Pf (Figure 3C). At the species level, *Purpureocillium_lilacinum*, *Fusarium_solani*, *Alternaria_alternata*, *Fusarium_equiseti*, *Scedosporium_apiospermum*, *Orbilia_oligospora*, *Ophiostoma_ulmi*, *Fusarium_tricinctum*, *Pyrenophora_teres* and *Fusarium_acuminatum* had higher RA in Pn than Zm, Nt and Pf, while *Trichoderma_asperellum* had lower RA in Pn than Zm, Nt and Pf (Figure 3D).

Using LefSe to identify differential fungal taxa (LDA ≥ 3) in SPP from the five treatment groups, it was found that a total of 17 fungal taxa exhibited significant differences. Among them, *Ophiostomatales*, *Ophiostomataceae*, *Ophiostoma*, *Ophiostoma_ulmi*, *Cladobotryum* and *Cladobotryum_mycophilum* taxa were significantly enriched in CK; *Talaromyces_ purpureogenus* was significantly enriched in Zm; *Mortierellomycetes*, *Apiotrichum*, *Mortierellales* and *Mortierellaceae* taxa were significantly enriched in Nt; and *Synchytriales*, *Synchytriaceae* and *Synchytrium*, *Synchytrium_taraxaci*, *Chytridiomycota*, and *Chytridiomycetes* taxa were significantly enriched in Pf (Figure 3E).

### 3.5 Potential functional pathways of soil microbial communities in SPP with added root exudates

**Figure 4:**
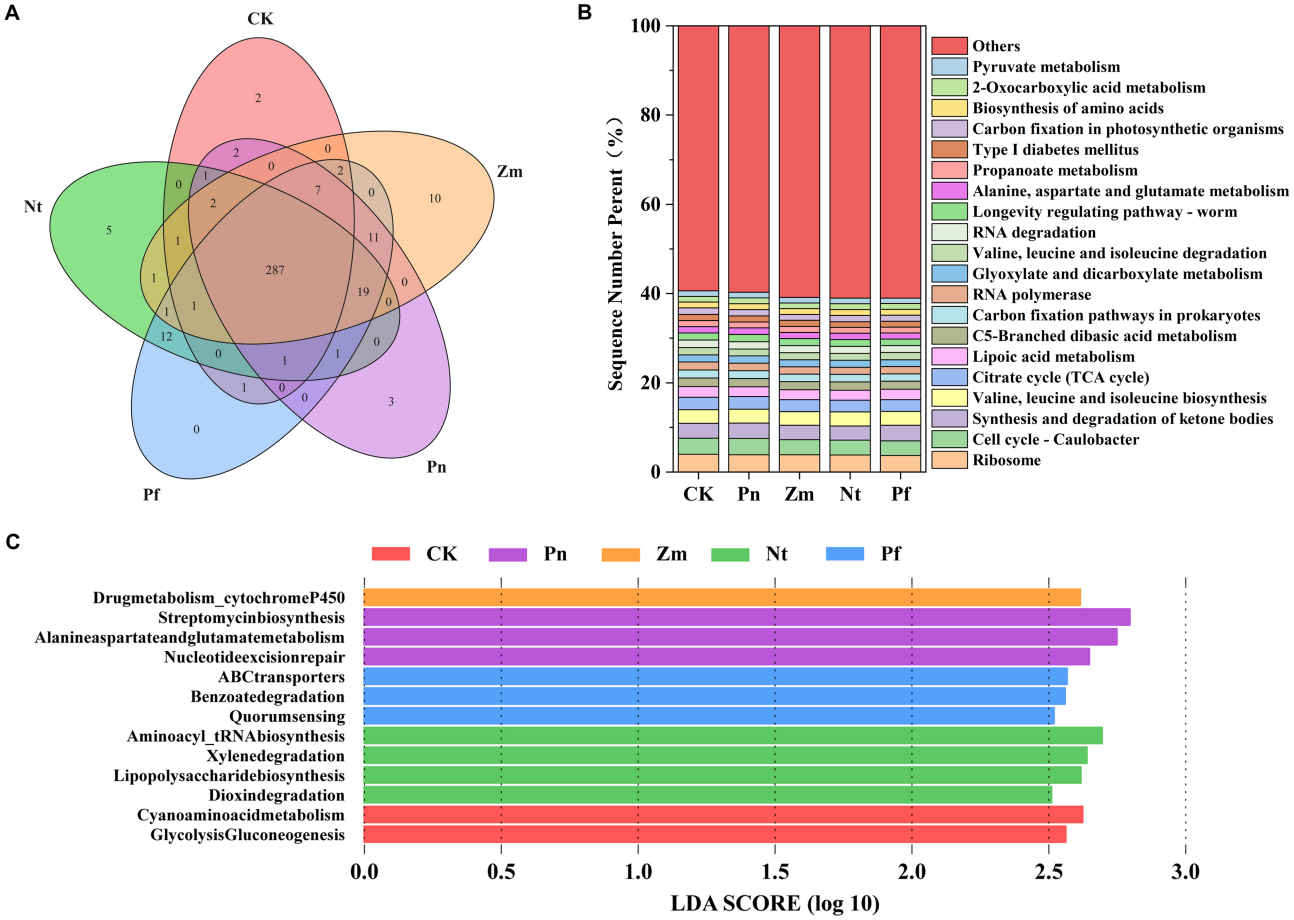
Effects of different root exudates on the microbial level 3 KEGG pathway in SPP. **(A)** Venn diagram. **(B)** Relative abundance plot. **(C)** Level 3 significantly different KEGG pathways based on LEfSe analysis (LDA ≥2.5).

Based on functional annotation of non-redundant genes based on the KEGG functional database, we compared the RA of potentially functional genes in SPP after different treatments. A total of 20,120 KEGG pathway-related genes were detected in all macrogenomes (Supplementary Table S9). A total of 307 level 3 KEGG pathways were annotated from the treated SPP. Among them, 287 pathways were common in CK, Pn, Zm, Nt, and Pf, and 2, 3, 10, 5, and 0 pathways were unique to CK, Pn, Zm, Nt and Pf, respectively (Figure 4A).

Among the level 3 KEGG annotated genes, the major categories were Ribosome (3.85%), Cell cycle-Caulobacter (3.45%), Synthesis and degradation of ketone bodies (3.33%), Valine, leucine and isoleucine biosynthesis (3.11%), Citrate cycle (TCAcycle) (2.70%) and Lipoic acid metabolism (2.27%), which accounted for 18.72% of the total gene abundance (Figure 4B). Among them, the first two groups belonged to genetic information and cellular processes, while the last four groups belonged to metabolism. In addition, among the first 20 categories, Pn increased Cell cycle-Caulobacter, Citrate cycle (TCA cycle), RNA polymerase, Glyoxylate and dicarboxylate compared to CK metabolism, RNA degradation, Longevity regulating-worm, Alanine, aspartate and glutamate metabolism and Carbon fixation in photosynthetic organisms, whereas the opposite was true for Zm, Nt and Pf (Figure 4B).

LEfSe analysis of all level 3 KEGG pathways revealed a total of 13 differential level 3 KEGG pathways (LDA ≥ 2.5) identified. Among them, the differential pathways in CK were Glycolysis/Gluconeogenesis, Cyanoamino acid metabolism; the differential pathways in Pn were Nucleotide excision repair, Alanine, aspartate, glutamate metabolism, Streptomycin biosynthesis; the differential pathway in Zm was Drug metabolism_cytochrome P450; the differential pathways in Nt were Dioxin degradatio, Lipopolysaccharide biosynthesis, Xylene degradation, Aminoacyl_tRNA biosynthesis; and Quorum sensing, Benzoate degradation, ABC transporters were differential pathways in Pf (Figure 4C).

### 3.6 Effects of root exudates and soil enzyme activities on microbial communities

**Figure 5:**
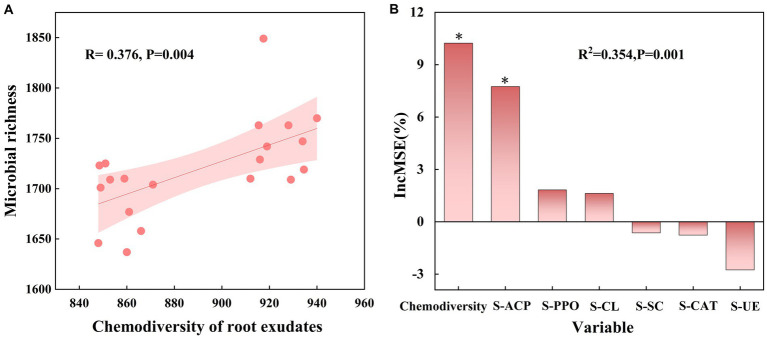
Associations between root exudates and microorganisms. **(A)** Correlations between chemodiversity of root exudates and biodiversity of microbiome. **(B)** Random forest model determining the key factors affecting the biodiversity of microbiome in SPP. Lines represent the least squares regression fits, and shaded areas represent the 95% confidence intervals. The importance of each predictor was determined by assessing the decrease in prediction accuracy [that is, the increase in the mean square error (%IncMSE) between observations and predictions] when the data for the predictor was randomly permuted. This decrease was averaged over all trees to produce the final measure of importance. ∗, ∗∗, and ∗∗∗ represent significant %IncMSE at *p* < 0.05, 0.01, and 0.001, respectively.

Soil enzymes play a role in various biochemical processes in soil and are also important indicators for characterizing soil microbial abundance. Measurement of six enzyme activities under different treatments showed that Pn significantly increased soil catalase activity (S-CAT), Nt significantly increased soil polyphenol oxidase activity (S-PPO), and Pf increased soil cellulase activity (S-CL), soil sucrase activity (S-SC), and soil polyphenol oxidase activity (S-PPO), compared to CK (Supplementary Figure S4). Linear regression analysis revealed a significant positive correlation (*r* = 0.376, *p* = 0.004) between chemicaldiversity of root exudates and microbial diversity (Figure 5A). Further random forest modeling showed that hemicaldiversity of root exudates (%IncMSE = 10.650, *p* < 0.01) had a greater effect on microbial diversity than soil enzyme activity (Figure 5B). These results indicate a strong relationship between chemicaldiversity of root exudates and microbial diversity.

### 3.7 Interrelationships between root exudates and soil microorganisms in SPP

**Figure 6:**
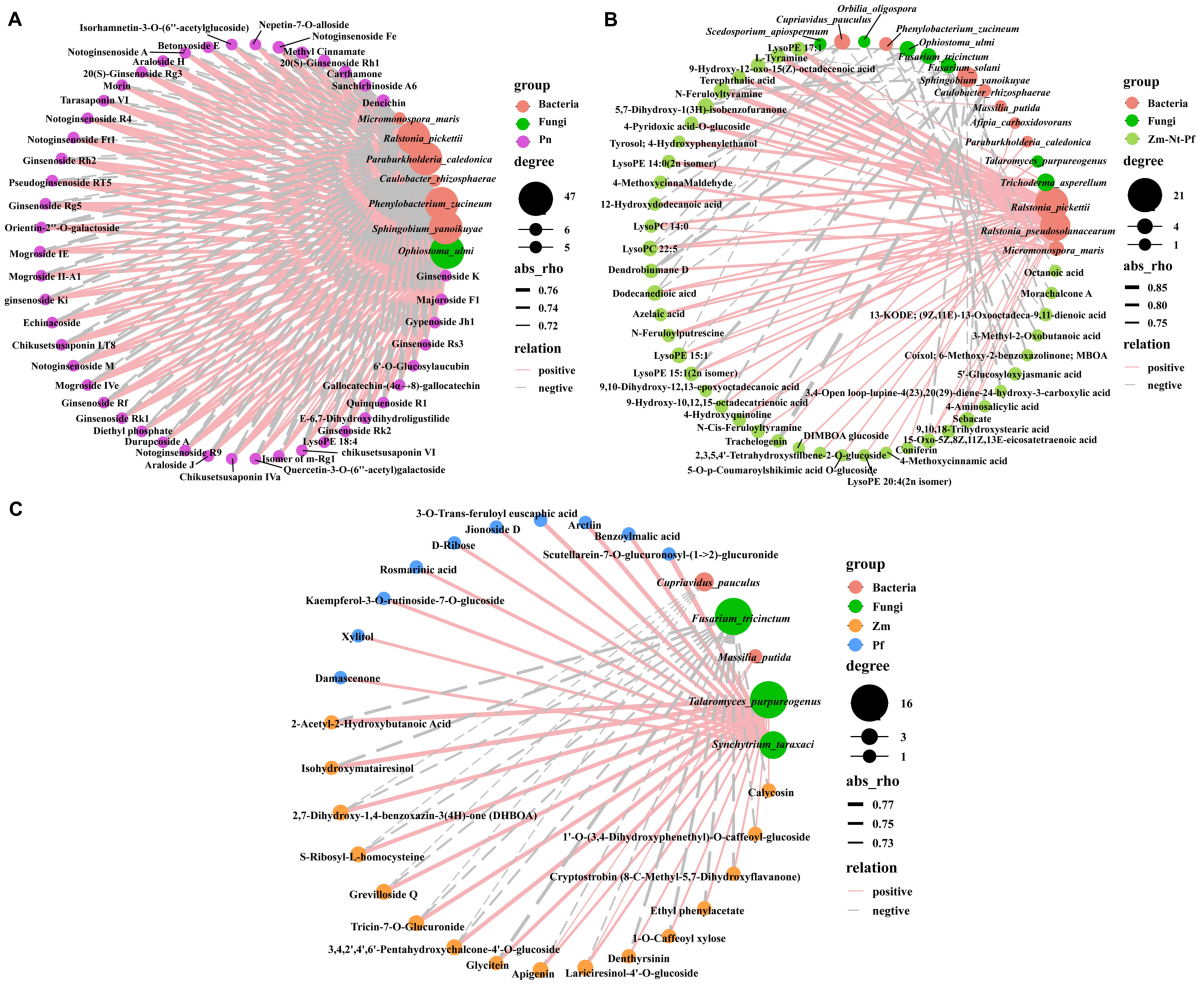
Correlation analysis between root exudates with bacteria and fungi. **(A)** Correlation between unique root exudates of Pn with bacteria and fungi. **(B)** Correlation between common root exudates of Zm, Nt, and Pf with bacteria and fungi. **(C)** Correlation between unique root exudates of Zm, Nt and Pf with bacteria and fungi. Red circles indicate bacteria, green circles indicate fungi, solid lines indicate positive correlations, dashed lines indicate negative correlations, and the thickness of the line indicates the strength of the correlation.

We used Spearman’s correlation analysis (*p* < 0.01) to reveal the correlation between DAMs and differential microbial species. All 47 unique DAMs identified in Pn, including terpenoids (32), flavonoids (7), and phenolic acids (3), others (2), amino acid derivatives (1), organic acids (1) and lipids (1) (Figure 1E, Supplementary Figure S2A, and Supplementary Table S3), were positively correlated with bacteria (*Phenylobacterium_zucineum* and *Sphingobium_yanoikuyae*) and fungi (*Ophiostoma_ulmi*), whereas they were negatively correlated with bacteria (*Paraburkholderia_caledonica* and *Ralstonia_pickettii*) (Figure 6A). Surprisingly, these 5 microbial strains that correlated with unique DAMs in Pn had opposite correlations with common DAMs in Zm, Nt and Pf (Figure 6B). Among them, organic acids (3-Methyl-2-Oxobutanoic acid, Sebacate) and flavonoid (Morachalcone A) were negatively correlated with *Phenylobacterium_zucineum*; lipids (5) were positively correlated with *Sphingobium_yanoikuyae*; phenolic acids (4-MethoxycinnaMaldehyde, 5′-Glucosyloxyjasmanic acid) and lipids (LysoPE 17:1) were positively correlated with *Ophiostoma_ulmi*, while lignans and coumarins (Trachelogenin) were positively correlated with *Paraburkholderia_caledonica*, and lipids (10) and alkaloids (5) were negatively correlated with *Ralstonia_pickettii* (Figure 6B). These results suggest that common root exudates of Zm, Nt and Pf were able to invert the abundance of microbial strains regulated by unique root exudates of Pn.

Interestingly, we also found that 18 common DAMs in Zm, Nt and Pf, including lipids (9-Hydroxy-12-oxo-15(Z)-octadecenoic acid, LysoPE 15:1, LysoPE 15:1(2n isomer), Dodecanedioic acid, 15-Oxo-5Z,8Z,11Z,13E-eicosatetraenoic acid, 9,10,18-Trihydroxystearic acid, LysoPE 14:0(2n isomer), LysoPE 20:4(2n isomer)), alkaloids (N-Feruloylputrescine, L-Tyramine, 4-Hydroxyquinoline, N-Feruloyltyramine, N-Cis-Feruloyltyramine), phenolic acids (5,7-Dihydroxy-1(3H)-isobenzofuranone, Terephthalic acid), organic acids (Azelaic, Sebacate), and others (4-Pyridoxic acid-O-glucoside) were positively correlated with the bacteria (*Ralstonia_pseudosolanacearum*) (Figure 6B). In addition, all 16 unique DAMs in Zm were positively associated with fungi (*Talaromyces_purpureogenus*) and negatively associated with fungi (*Fusarium_tricinctum*), and all 10 unique DAMs in Pf were positively associated with fungi (*Synchytrium_taraxaci*), while no associated microorganisms were found for all 17 unique DAMs in Nt (Figure 6C).

### 3.8 Interrelationships between root exudates and soil microbial functional pathways in SPP

**Figure 7:**
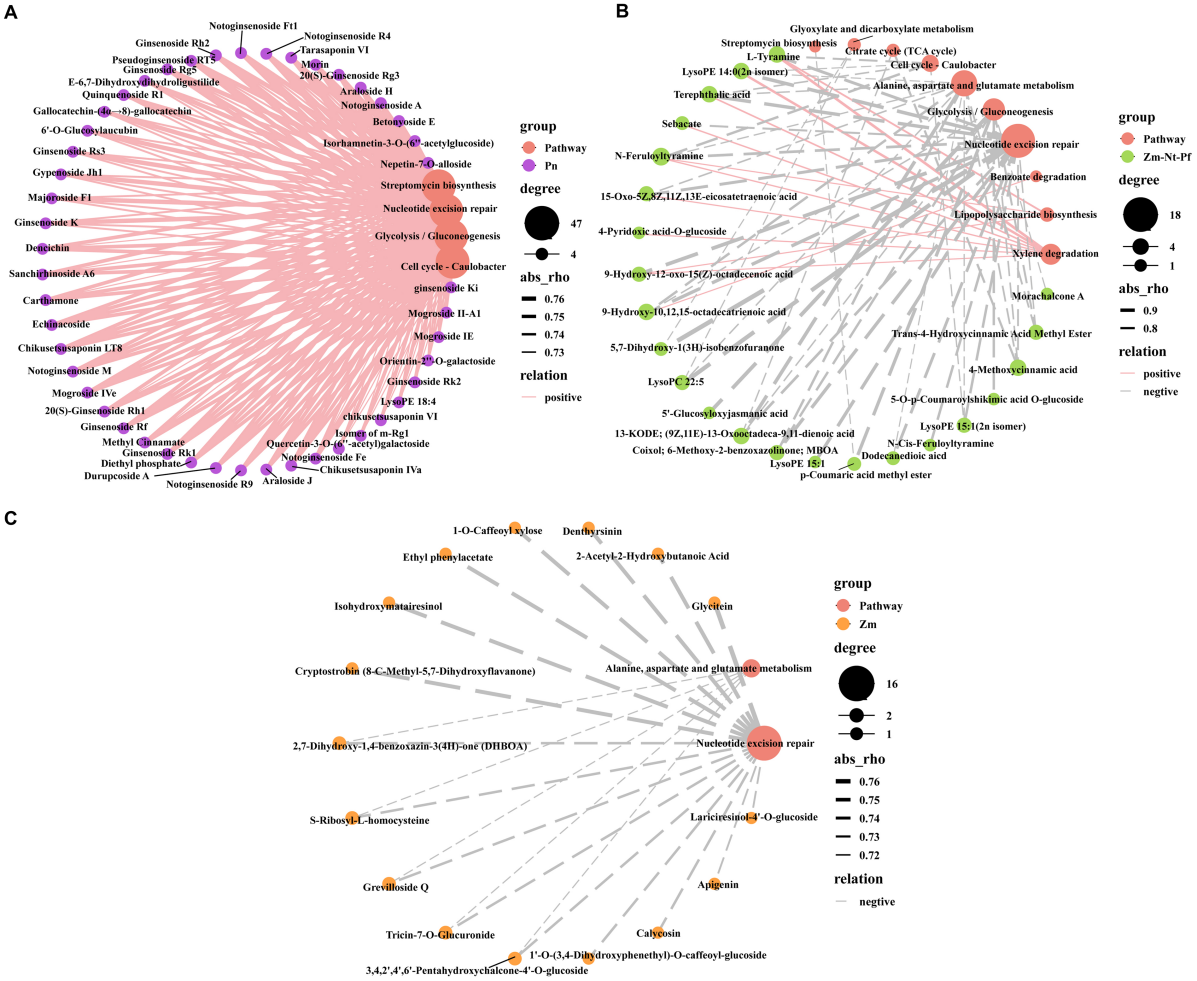
Correlation analysis between root exudates and level 3 KEGG pathway. **(A)** Correlation between unique root exudates of Pn and level 3 KEGG pathway. **(B)** Correlation between common root exudates of Zm, Nt, and Pf and level 3 KEGG pathway. **(C)** Correlation between unique root exudates of Zm, Nt and Pf and level 3 KEGG pathway. Solid lines indicate positive correlations, dashed lines indicate negative correlations, and the thickness of the line indicates the strength of the correlation.

Spearman’s correlation analysis (*p* < 0.01) between DAMs and functional pathways of differential microbial species showed that all 47 unique DAMs identified in Pn (Figure 1E, Supplementary Figure S2A, and Supplementary Table S3), were positively correlated with Nucleotide excision repair, Streptomycin biosynthesis, Cell cycle- Caulobacter and Glycolysis/Gluconeogenesis pathways (Figure 7A), while these pathways were negatively correlated with common DAMs in Zm, Nt and Pf (Figure 7B). Among them, common 18 DAMs in Zm, Nt and Pf, including lipids (9), alkaloids (4), phenolic acids (3) and organic acids (2) were negatively correlated with Nucleotide excision repair pathway; phenolic acid (p-Coumaric acid methyl ester) was negatively correlated with Streptomycin biosynthesis pathway; alkaloids (L-Tyramine, N-Cis-Feruloyltyramine), lipids [LysoPE 14:0 (2n isomer), LysoPE 15:1 (2n isomer)] and phenolic acid (Terephthalic acid) were negatively correlated with Cell cycle- Caulobacter pathway; phenolic acids [5′-Glucosyloxyjasmanic acid, 5-O-p-Coumaroylshikimic acid O-glucoside, p-Coumaric acid methyl ester (4-Methoxycinnamic acid), lipids (13-KODE; (9Z,11E)-13-Oxooctadeca-9,11-dienoic acid, 9-Hydroxy-10,12,15-octadecatrienoic acid), flavonoids (Morachalcone A), alkaloids (Coixol; 6-Methoxy-2-benzoxazolinone; MBOA) and organic acids (Trans-4-Hydroxycinnamic Acid Methyl Ester)] were negatively associated with Glycolysis/Gluconeogenesis pathway (Figure 7B).

We also found that 18 common DAMs in Zm, Nt and Pf, including lipids (7), phenolic acids (2), alkaloids (2), organic acids (1) were negatively correlated with the Alanine, aspartate and glutamate metabolism pathway; 8 common DAMs in Zm, Nt and Pf, including lipids (3), alkaloids (2), phenolic acids (1), organic acids (1) and others (1) were positively correlated with Xylene degradation pathway (Figure 7B). In addition, all 17 unique DAMs in Zm were negatively correlated with the Nucleotide excision repair pathway, and 5 unique DAMs including flavonoids (Tricin-7-O-Glucuronide, 3,4,2′,4′,6’-Pentahydroxychalcone-4’-O-glucoside), alkaloids [2,7-Dihydroxy-1,4-benzoxazin-3(4H)-one (DHBOA)], amino acids and their derivatives (S-Ribosyl-L-homocysteine), and Others (Grevilloside Q) were also negatively correlated with the Alanine, aspartate and glutamate metabolism pathway (Figure 7C). These results suggest that these enriched or inhibited functional pathways are highly relevant to the changing microbial community.

## 4 Discussion

In this study, we described the effects of root exudates of Pn, Zm, Nt and Pf on the composition and function of microbial communities in SPP. Unlike previous rotations of *P. notoginseng* with different crops carried out in different plots (Wang F. et al., 2022), the static soil culture we used ensured homogeneity of root exudates interacting with soil microbes as well as easy identification of early and rapidly responding microflora. Our study showed that there was a high chemical diversity of root exudates from different plant species (Figure 1D), and that this diversity was significantly and positively correlated with microbial diversity (Figure 5A). In a previous study, chemodiversity of rhizodeposits had a greater effect on active bacterial biodiversity than soil physicochemical properties (Li T. et al., 2022). This may be ascribed to higher resource diversity in root exudates which can provide wider habitat niche breadth for organisms, therefore promoting higher biodiversity (Tanentzap et al., 2019).

Bacteria, as an important flora in the soil, its composition and diversity play an important role in the healthy growth of *P. notoginseng*. Here, Proteobacteria, Acidobacteria and Actinobacteria were the major bacterial phyla found in all soil samples, accounting for 97.52% of the total bacterial taxa (Figure 2C). Previous study also confirmed that the bacterial taxa in *P. notoginseng* soil, either cropland planting or understory planting, were also mainly dominated by Proteobacteria (34.5%), Acidobacteria (28.6%) and Actinobacteria (10.5%) (Kui et al., 2021). However, we found that Zm and Pf increased the RA of Actinobacteria, whereas Pn and Nt decreased its RA (Figure 2C). Actinobacteria typically utilize flavonoids as substrates to produce diverse secondary metabolites, resulting in rare and highly active biofunctional derivatives (Bai et al., 2014; Wang et al., 2020). This is explained by the fact that the unique root exudates in Zm and Pf are mainly flavonoids and phenolic acids (Supplementary Figures S2B,D). Consistent with the study results, Pn decreased RA of *Ascomycetes* and *Actinomycetes*, while Zm, Nt and PF increased their RA at the species level (Figure 2D). Proteobacterias, Actinobacterias and Bacteroidetes increased significantly in rotations with *Coix lacryma-jobi*, *Oryza sative*, *N. tabacum*, *Capsicum annuum* and *Zingiber officinale*, respectively, compared with continuous cropping *P. notoginseng* (Wang F. et al., 2022). Interestingly, among the significantly enriched bacterial taxa (LDA ≥ 3.5), *Sphingomonadaceae*, *Sphingobium* and *Sphingobium_yanoikuyae* were significantly enriched in Pn, whose predominant core genera in cropland planting soil of *P. notoginseng* in previous study also included *Sphingomonas*, but the exact role is not clear (Kui et al., 2021).

Increased abundance of fungi in the soil was the main cause of pathogenesis in *P. notoginseng* plants. At the level of fungal phyla, Ascomycota, Mucoromycota and Basidiomycota were the dominant fungi in *P. notoginseng* planting soil (Figure 3C), which is agreement well with the results of previous studies (Kui et al., 2021). At the level of ascomycete species, the RA of *Purpureocillium_lilacinum*, *Fusarium_solani*, *Alternaria_alternata*, *Fusarium_equiseti*, *Scedosporium_apiospermum*, *Orbilia_ oligospora*, *Ophiostoma_ulmi*, *Fusarium_tricinctum*, *Pyrenophora_teres* and *Fusarium_acuminatum* were increased in Pn (Figure 3C). Previous studies have shown that the RA of ascomycetes increased significantly with the growth of *P. notoginseng*, while the proportion of ascomycetes in the rotation of other crops decreased to varying degrees (Luo et al., 2019; Wang F. et al., 2022), suggesting that ascomycetes may be the causative organisms leading to CCO in *P. notoginseng*. In addition, *F. oxysporum* is an important soil-borne disease that mainly causes the occurrence of crop wilt and is recognized as one of the three major soil-borne pathogenic fungi in the world (Zuriegat et al., 2021). Numerous studies have also shown that *F. oxysporum* causes root rot in *P. notoginseng* during cultivation (Dong et al., 2016; Fan et al., 2016; Li T. et al., 2022). We found that the RA of *F. oxysporum* was the highest in all treatments (Figure 3D), suggesting that even after 2 years of fallow, it could still exist as a dominant species in the soil. However, we also found that the RA of *F. oxysporum* in Pn was lower than that in the CK and other treatments (Figure 3D), and this contradictory result may imply that its recognition of *P. notoginseng* exudates is a long-term accumulation process.

Some compounds in root exudates affect the health of *P. notoginseng* by modulating the changes of bacteria and fungi in soil. In this study, all 47 unique DAMs in Pn were found to be positively correlated with bacteria (*Phenylobacterium_zucineum*, *Sphingobium_yanoikuyae*) and fungi (*Ophiostoma_ulmi*), whereas all common DAMs in Zm, Nt and Pf were negatively correlated with them (Figures 6A,B). Previous study reported that facultative intracellular *Phenylobacterium_zucineum* may have pathogenic relevance to humans and mammals (Zhang et al., 2007). Syn-RP4 consisting of four genera (*Dyadobacter*, *Sphingobacterium*, *Sphingobium* and *Sphingopyxis*) synergistically with the pathogenic fungus (*Ilyonectria destructans*) aggravates *P. notoginseng* root rot (Guo et al., 2022). *Ophiostoma ulmi* is the main pathogenic fungus responsible for widespread mortality of elm trees in Europe (Santini and Faccoli, 2015). In contrast, all unique DAMs in Pn were negatively correlated with bacteria (*Paraburkholderia_caledonica*, *Ralstonia_pickettii*), while all common DAMs in Zm Nt and Pf were positively correlated with them (Figures 6A,B). *Paraburkholderia* is a potential biocontrol agent with antimicrobial pro-biotic properties, which has been identified in the in the rhizosphere soil of *P. notoginseng* as a beneficial bacterial population capable of being significantly enriched by autotoxic saponins (Jeong et al., 2003; Kunakom and Eustáquio, 2019; Luo et al., 2021). *Ralstonia_pickettii* also has great biotechnological potential in the field of bioremediation and has been shown to be able to decompose toxic substances such as toluene and trichloroethylene (Ryan et al., 2007). In addition, we found that unique DAMs in Zm were positively correlated with the fungus (*Talaromyces_purpureogenu*s) and negatively correlated with the fungus (*Fusarium_tricinctum*) (Figure 6C). *Talaromyces purpurogenus* Q2 and *Talaromyces* spp., highly effective biocontrol strains isolated and characterized previously, were able to significantly inhibit pathogens (Abbas et al., 2021; Tian et al., 2021), whereas infection with *Fusarium_tricinctum* produces toxins that contaminate the grains resulting in high yield losses and reduced quality (Wang Y. et al., 2022). We also found a positive correlation between unique DAMs in Pf and the fungus (*Synchytrium_taraxaci*) (Figure 6C), which has not been reported yet. These results suggest that unique root exudates in Pn enriched potentially harmful microbiota and suppressed potentially beneficial microbiota, whereas common and unique root exudates in Zm, Nt and Pf ameliorated this situation.

Metabolic pathways enriched for root secretion release may lead to different physiological consequences and are important for soil health and plant growth. Unique DAMs in Pn enriched functional pathways of Nucleotide excision repair, Streptomycin biosynthesis, Cell cycle-Caulobacter and Glycolysis/Gluconeogenesis, whereas common DAMs in Zm, Nt and Pf reduced the enrichment of these functional pathways (Figures 7A,B). Similarly, unique DAMs in Zm also reduced the enrichment of the Nucleotide excision repair pathway (Figure 7C). In order to protect themselves from the severe consequences of DNA damage from multiple sources such as UV irradiation and environmental toxins, cells have evolved a multifunctional Nucleotide excision repair pathway, and this pathway has been found to be one of the major pathways for rhizosphere enrichment in continuous cropping peanuts (Li et al., 2018; Kraithong et al., 2021; D’souza et al., 2022). Streptomycin is commonly used as antibiotic which inhibits soil-borne pathogens to a large extent (Liu et al., 2015). Caulobacter was shown to model plant-microbe and microbe-microbe interactions in ecotypes (Berrios, 2022). Glycolysis/Gluconeogenesis is a key pathway for the production of energy and biomolecular synthesis intermediates (Gupta and Gupta, 2021). Thus, this suggests that unique root exudates in Pn inhibit the growth of beneficial bacteria by causing damage to them, and that there is also some antagonism between beneficial and pathogenic bacteria.

## 5 Conclusion

**Figure 8:**
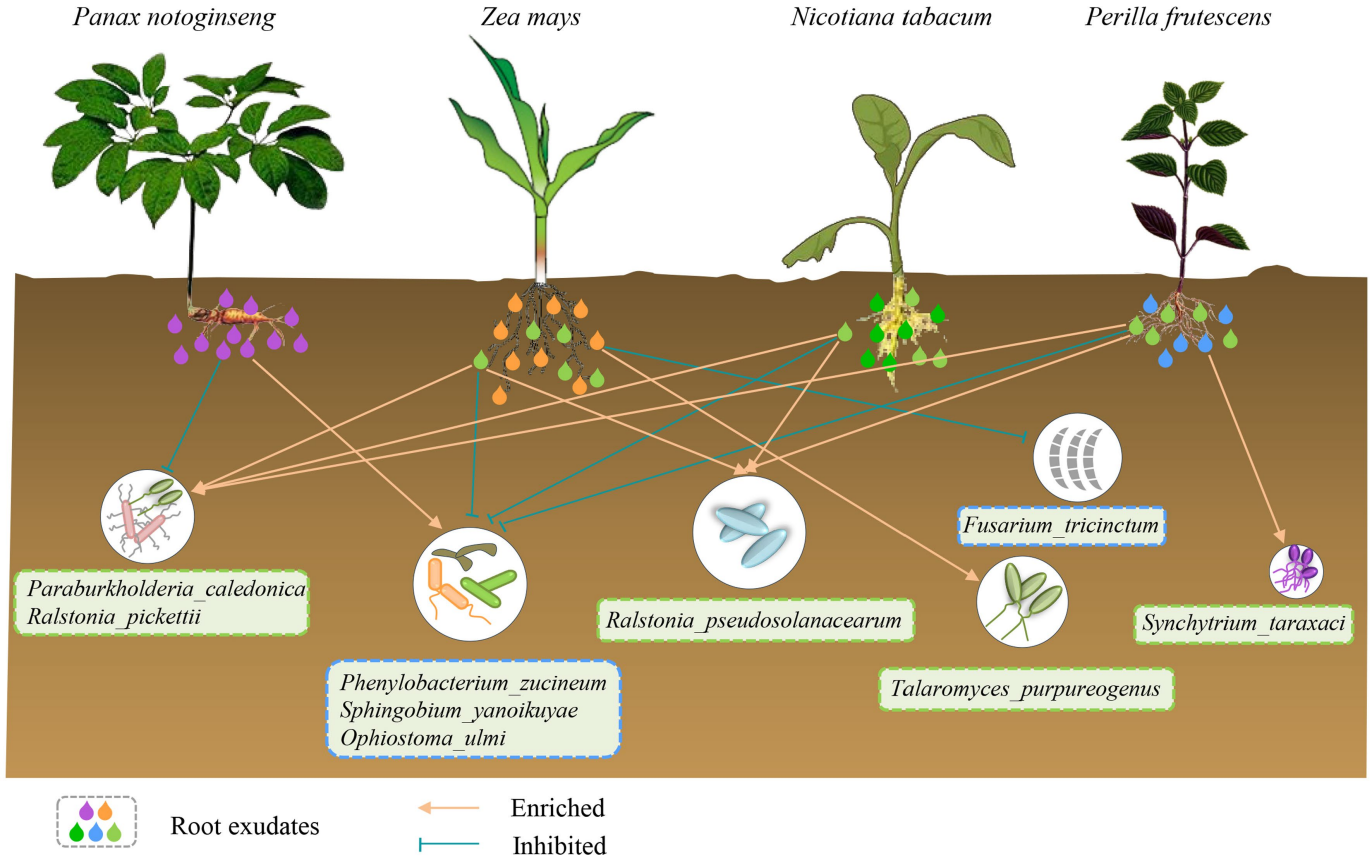
The schematic diagram illustrates the specific interactions of four plant species with microorganisms in the soil cultivated with Pn through root exudates. The specific root exudates of Pn enrich or inhibit some potentially harmful or beneficial strains in the soil of Pn, resulting in autotoxicity. However, the specific root exudates of Zm, Nt and Pf have the opposite effects. In addition, the specific root exudates of Zm can enrich potentially beneficial bacteria while inhibit potentially harmful bacteria to promote health. Also, Pf specific root exudates can enrich potentially beneficial bacteria to maintain their own health.

The chemical diversity of root exudates of *P. notoginseng*, *Z. mays*, *N. tabacum* and *P. frutescens* differed significantly and had a strong influence on the microbial diversity of soils where *P. notoginseng* had been planted. Unique root exudates of *P. notoginseng* recruited potentially harmful flora and inhibited potentially beneficial flora in the soil, which could be reversed by root exudates common in *Z. mays*, *N. tabacum* and *P. frutescens* (Figure 8).

## Data availability statement

The datasets presented in this study can be found in online repositories. The names of the repository/repositories and accession number(s) can be found in the article/Supplementary material.

## Author contributions

HS: Data curation, Methodology, Visualization, Writing – review & editing. JY: Data curation, Visualization, Writing – original draft. QL: Writing – review & editing. CP: Writing – review & editing. ZS: Writing – review & editing. HY: Writing – review & editing. YL: Writing – review & editing. CL: Writing – review & editing. WF: Supervision, Writing – original draft.
