# Supplementary figures and images for: Diversity and correlation analysis of different root exudates on the regulation of microbial structure and function in soil planted with Panax notoginseng
Source: Front Microbiol. 2023 Dec 6;14:1282689. doi: 10.3389/fmicb.2023.1282689 (PMC10731274; doi:10.3389/fmicb.2023.1282689)

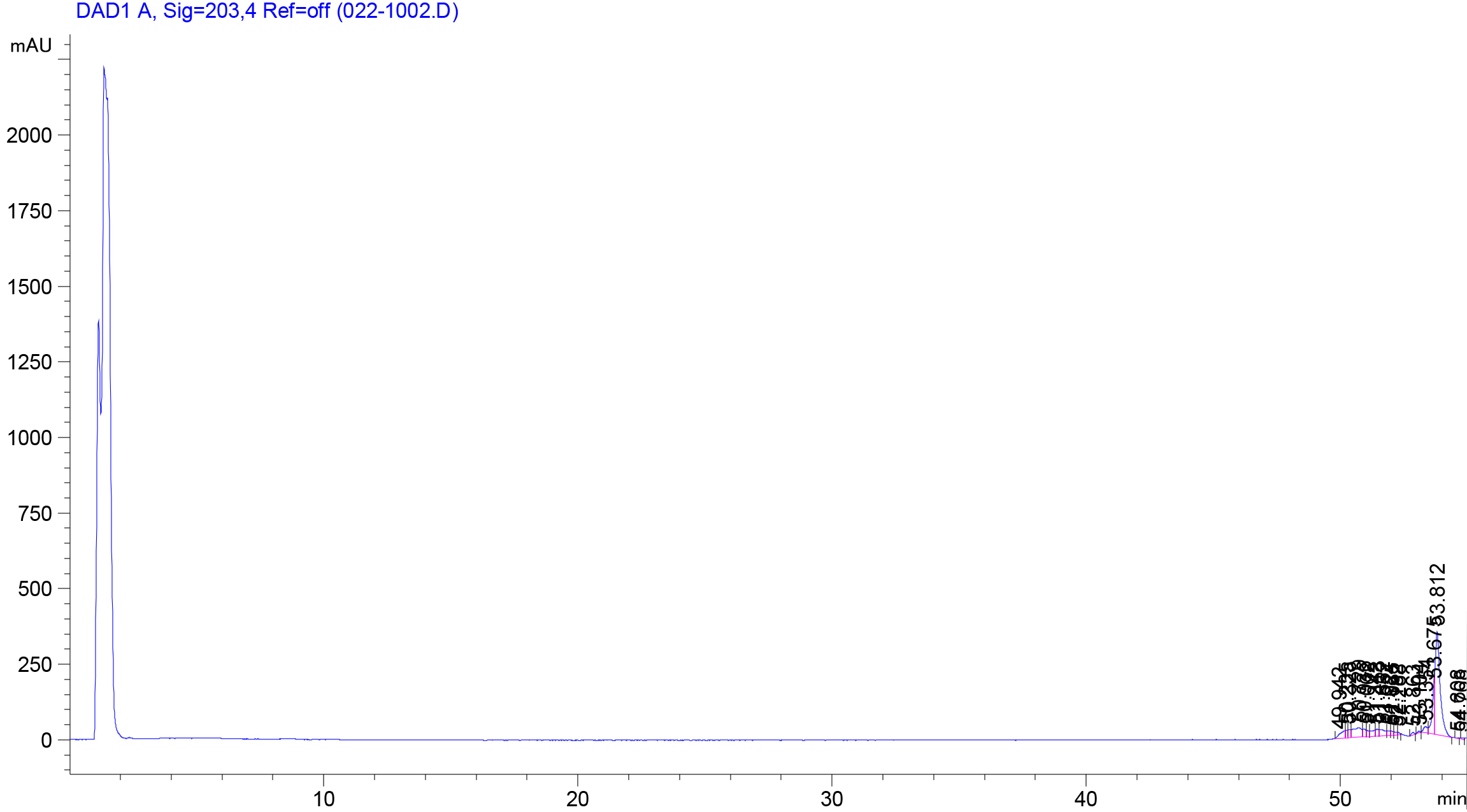

Supplement: Supplementary file 1 [file Image_1.JPEG]

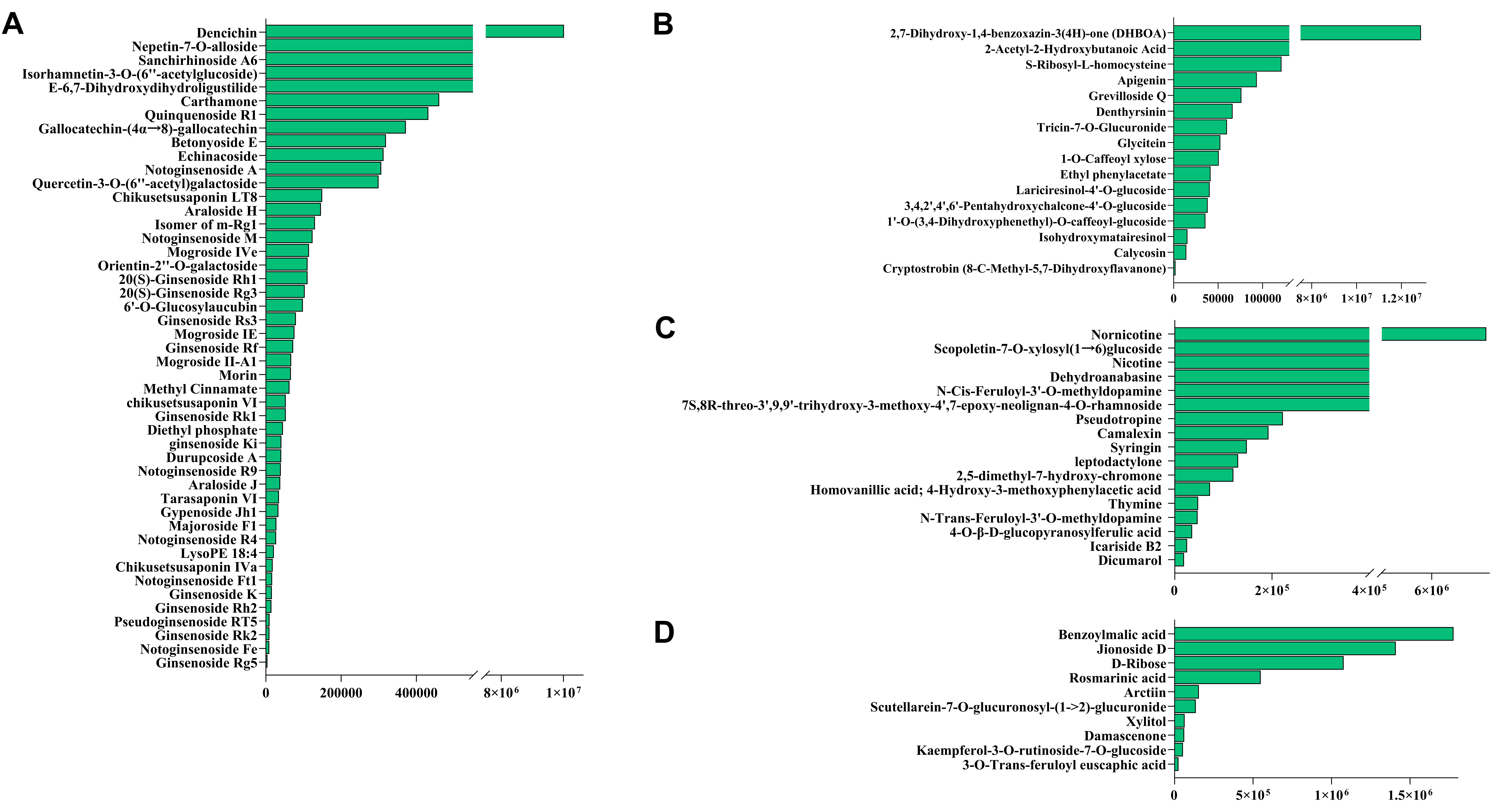

Supplement: Supplementary file 2 [file Image_2.JPEG]

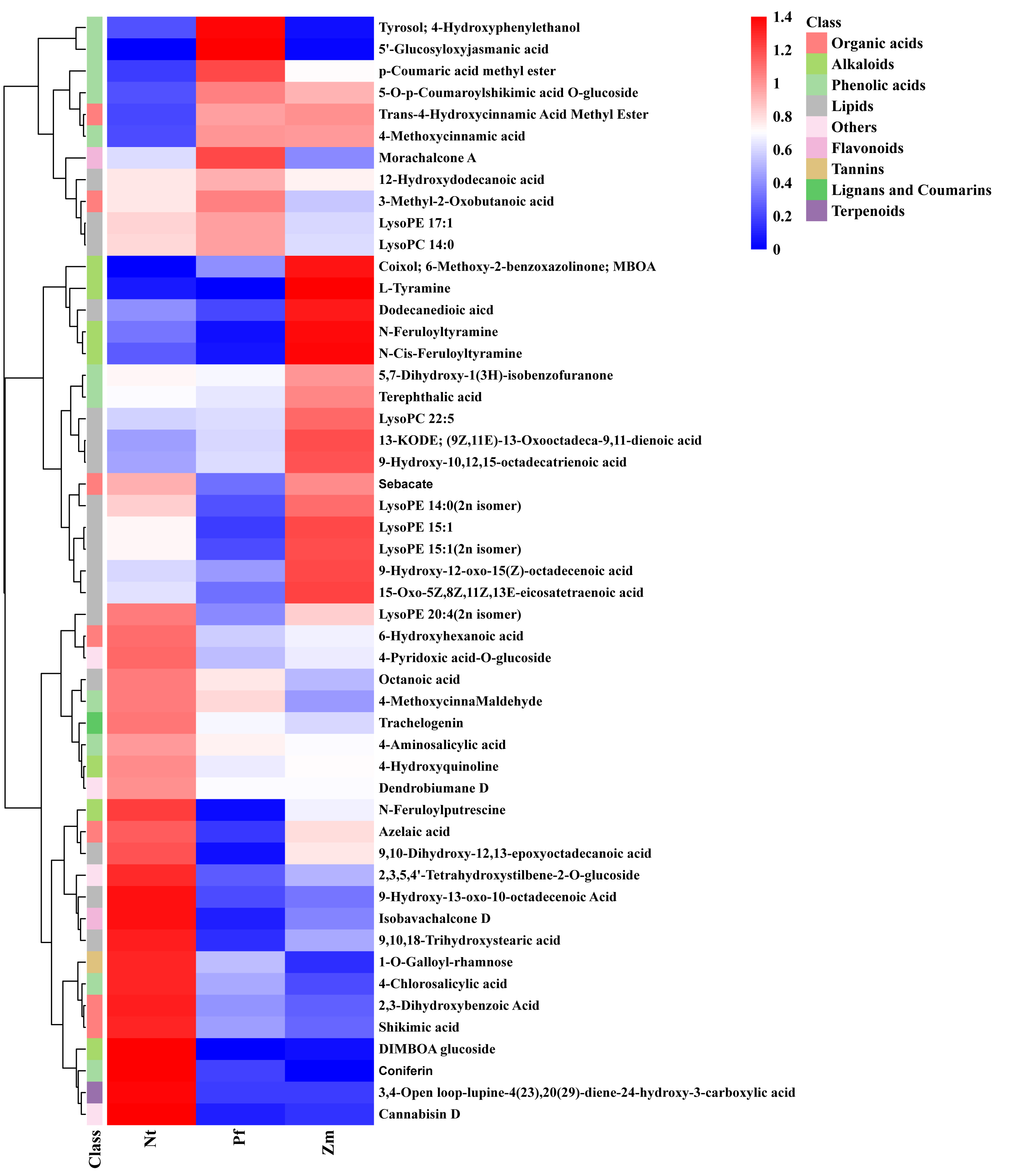

Supplement: Supplementary file 3 [file Image_3.JPEG]
